# Antidiabetic Drug Efficacy in Reduction of Mortality during the COVID-19 Pandemic

**DOI:** 10.3390/medicina59101810

**Published:** 2023-10-11

**Authors:** Daniel Gonikman, Dmitrijs Kustovs

**Affiliations:** 1 Student of Faculty of Medicine, Riga Stradins University, LV-1007 Riga, Latvia; 2 Department of Pharmacology, Riga Stradins University, LV-1007 Riga, Latvia; dmitrijs.kustovs@rsu.lv

**Keywords:** COVID-19, diabetes mellitus, mortality

## Abstract

*Background and Objectives*: The COVID-19 pandemic caused by the Coronavirus SARS-CoV-2 is a complex challenge for the existing scientific and medical landscape. It is an ongoing public health crisis, with over 245,373,039 confirmed cases globally, including 4,979,421 deaths as of 29 October 2021. Exploring molecular mechanisms correlated with the disease’s severity has demonstrated significant factors of immune compromise, noted in diabetic patients with SARS-CoV-2 infections. Among diabetics, the altered function of the immune system allows for better penetration of the virus into epithelial cells, increased viral binding affinity due to hyperglycemia, reduced T cell function, decreased viral clearance, high risks of cytokine storm, and hyper-inflammatory responses, altogether increasing the susceptibility of these patients to an extreme COVID-19 disease course. *Materials and Methods:* This research involved a systematic literature search among various databases comprising PubMed and Google Scholar in determining credible studies about the effects of antidiabetic drugs on the high mortality rates among diabetic patients infected with COVID-19. The primary search found 103 results. Duplicated results, non-pertinent articles, and the unavailability of full text were excluded. Finally, we included 74 articles in our review. The inclusion criteria included articles published during 2020–2023, studies that reported a low risk of bias, and articles published in English. Exclusion criteria included studies published in non-peer-reviewed sources, such as conference abstracts, thesis papers, or non-academic publications. *Results:* Among the studied anti-diabetic drugs, Metformin, the Glucagon-like peptide 1 receptor agonist (GLP-1RA), and Sodium-glucose co-transporter 2 inhibitors (SGLT-2i) have demonstrated decreased mortality rates among diabetic patients infected with COVID-19. Insulin and Dipeptidyl peptidase 4 inhibitors (DPP-4i) have demonstrated increased mortality rates, while Sulfonylureas, Thiazolidinedione (TZD), and Alpha-glucosidase inhibitors (AGI) have demonstrated mortality-neutral results.

## 1. Introduction

Diabetes is a global disease that poses major health risks [1] as its prevalence has significantly increased in the last two decades [2]. Diabetes has escalating trends: by 1985, 30 million individuals were diagnosed with diabetes globally, increasing in 2010 to 285 million with the global estimate of affected individuals in 2022 standing at 530 million, with an approximation that by 2045, close to 700 million individuals will be affected [3]. These figures indicate that diabetes is causing high mortality rates globally, hence the need for requisite interventions to handle the growing number of affected individuals worldwide [4]. Diabetes leads to non-traumatic lower extremity amputations, end-stage renal disease, and adult-onset blindness [5]. These complications cause severe disabilities and chronic disorders [2] among affected individuals. Diabetes causes major impairments in the immune system function, including decreased cytokines production and thus, suppressed immune response against invading pathogens [6], decreased leukocytic activity, defective leukocyte recruitment [7], and neutrophil dysfunction [6]. Zhu et al. (2020) explain that poor glycemic control predicts an increased need for medications and hospitalizations, and increased mortality [8]. Diabetes is also a leading risk factor for a severe COVID-19 infection course, among other risk factors such as age >65 years old, chronic pulmonary disease, male sex, cancer, smoking history, cardiovascular disease, and unvaccinated patients [9,10]. Anti-diabetic drugs have been proposed to treat diabetic patients with a COVID-19 infection because of their variable effects on the cellular and molecular levels [11]. First, their ability to decrease cytokine production [1,12,13,14] reduces cytokine levels in vascular cells [11], preventing endothelial injury and reducing the risk of thromboembolism [15] and microvascular damage [1]. This ability also alleviates the cytokine storm and, thus, decreases lung injury which is the hallmark of a severe COVID-19 infection [11]. Additionally, by decreasing gut inflammation, anti-diabetic drugs improve glucose metabolism [5]. Some studies found that Metformin is capable of reaching lysosomes, increasing their pH [11,16]; this prevents viral entry into the cells and blocks its replication as viruses need an acidic environment to function [11]. Metformin also protects against secondary fibrosis in the lungs [1]; in the past, it has been successfully used as an anti-influenza drug due to its anti-inflammatory properties [11,17]. In this review, we will describe these mechanisms more profoundly to better explain the possible effect of anti-diabetic drugs on mortality reduction among COVID-19 patients.

With the rapidly growing number of individuals diagnosed with diabetes worldwide [18], their increased risk of developing severe complications of the COVID-19 infection [1] as well as the emergence of new COVID-19 strains [10], and the findings suggesting that diabetes is a major risk factor for a severe COVID-19 infection course [8], we aim to describe the possible anti-viral mechanisms of anti-diabetic drugs, their efficacy in decreasing mortality rates, their possible side effects, and previous studies’ findings. Our study results may offer valuable insights for healthcare professionals when selecting suitable glucose-lowering treatments for diabetics with COVID-19, reducing hospital admissions and the duration of in-hospital stay, as well as helping with better treatment strategies in future viral outbreaks that might progress to pandemics. Additionally, by finding that certain antidiabetic drugs are effective in reducing mortality among diabetics with a COVID-19 infection, we would be able to prioritize these drugs for use in these specific patient populations, potentially helping allocate limited resources more effectively as COVID-19 infections have strained healthcare resources worldwide [19]. Furthermore, antidiabetic drugs are readily available [20] and widely prescribed [21,22,23], so repurposing them for COVID-19 treatment could provide an accessible option for the treatment of diabetics with a COVID-19 infection.

## 2. Materials and Methods

This research involved a systematic literature search among various databases comprising PubMed, Google Scholar, and Embase in determining credible studies considering the effects of antidiabetic drugs on high mortality rates among diabetic patients infected with COVID-19. The search strategy was conducted on studies from 2020 to 2023 to ensure the inclusion of current results during the significant stages of the pandemic. The keywords used in this search entailed combinations of terms such as “mortality”, “diabetes”, “antidiabetic drugs”, and “COVID-19”. Citations were downloaded into Endnote X8 and duplicates were removed.

## 3. Results

- Insulin

**Figure 1:**
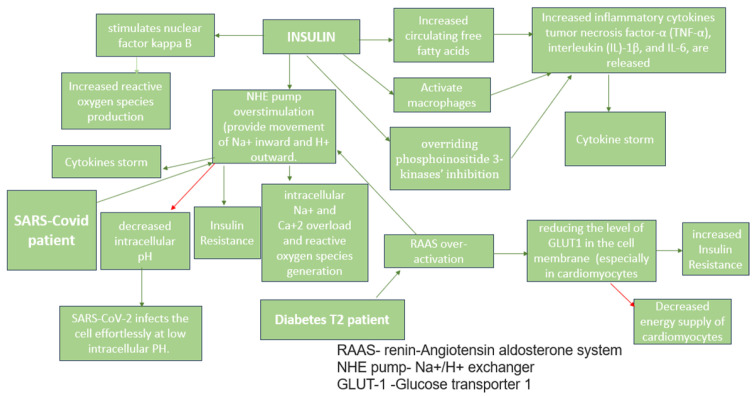
Pro-Viral Effects of Insulin [26,27]—The red arrows indicate inhibition (decrease); green arrows indicate stimulation (increase).

Insulin therapy is a comprehensive tool for managing high blood glucose levels [1]. High blood glucose levels are associated with immune malfunctioning, hormonal changes such as increased catecholamines and glucocorticoid production [10], damage of blood vessels’ endothelium [1], increased inflammation, and viral binding and replication [24], thus, worsening the COVID-19 course [6]. Conversely, insulin might not effectively minimize mortality by maintaining normal blood glucose rates within the expected levels’ span [25], as it has variable effects (Figure 1): it increases the production of inflammatory cytokines, Tumor Necrosis Factor-a (TNF-a), interleukin IL-1B, and IL-6 through the nuclear factor kappa B pathway [25]; it increases the affinity of the SARS-CoV-2 spike protein and predisposes diabetic patients to a severe COVID-19 disease course [26]. Cumhur et al. (2023) assert that, in patients with type 2 DM, the hyperactive Renin–Angiotensin System (RAS) increases Na+/H+ exchanger activity (NHE), decreasing intracellular pH and predisposes the patients to a SARS-CoV-2 infection since it infects the cell effortlessly at a low intracellular pH [26]. Increased NHE activity also leads to insulin resistance and further worsens hyperglycemia [26]. A meta-analysis done by Nguyen et al. (2022) included 61 studies with 3,061,584 individuals and found insulin as a cause of increased mortality among diabetes T2 patients infected with SARS-CoV-2 (OR 1.70, 95% CI 1.33–2.19) [12].

- Metformin

**Figure 2:**
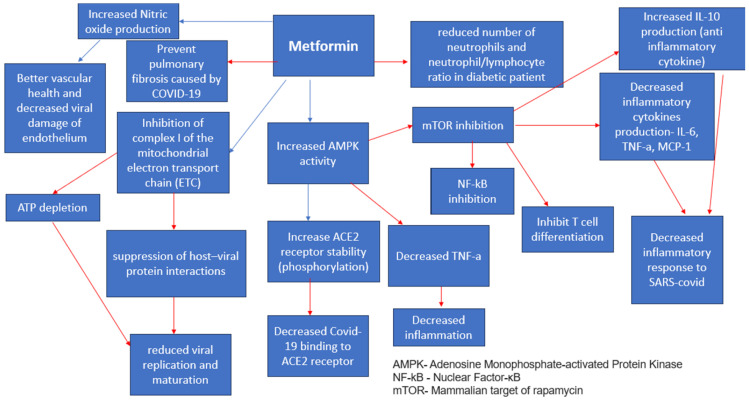
Metformin Anti-Viral Effects [4,31]. Red arrows indicate inhibition (decrease); blue arrows indicate stimulation (increase).

Metformin is a first-line drug for diabetes management [27,28]. It has several favorable effects that help manage diabetics with a COVID-19 infection [29] (Figure 2). It induces weight loss [30] and provides better glycemic control through decreasing insulin resistance [31]. Additionally, it has cellular-protecting properties independent of the prevailing blood glucose concentration [32]. At the liver level, metformin inhibits the mitochondrial respiratory chain [33], activating AMP protein kinase (AMPK) [29,34] and improving insulin sensitivity through effects on fat metabolism [4]. Additionally, it decreases cAMP, thus reducing the expression of gluconeogenic enzymes [33]. Furthermore, metformin inhibits fructose-1,6- bisphosphatase by a hepatic AMPK-independent mechanism [33]. It changes the composition of the microbiota [5], thus reducing inflammation. In addition, it downregulates the expression of genes encoding pro-inflammatory cytokine production [15], significantly decreasing excessive inflammatory responses and thus slowing disease progression [4]. Metformin also reduces neutrophil and macrophage infiltration in hyperoxia-induced lung damage [35]. The induction of autophagy by metformin also contributes to the elimination of pathogens [4]. Metformin exerts its effects mainly through the activation of the AMP-activated protein kinase (AMPK) pathway [31]. The AMPK functions as an important sensor of physiological energy levels [4,31], primarily responding to fluctuations in glucose availability. In times of elevated blood glucose levels in diabetic patients, the dynamics of AMPK signaling pathways undergo alterations [16], affecting cellular metabolism, growth, and proliferation [4]. When cellular energy levels are depleted, AMPK activation prompts a heightened glucose uptake in the skeletal muscle [4] and enhances fatty acid oxidation in adipose tissue [4]. The AMPK suppresses the activity of the mammalian target of the Rapamycin Kinase (mTOR) pathway [4,33] thus slowing down disease progression as mTOR is thought to take part in the metabolic syndrome and diabetes progression [33]. Metformin modulates catalase and superoxide dismutase levels [36], which are found in high levels in the serum of patients with a COVID-19 infection, thus preventing a cytokine storm [4]. It also decreases neutrophils and the neutrophil/lymphocyte ratio in diabetic patients [31]. Kamyshnyi et al. (2021) write about the AMPK ability to regulate the expression and stability of ACE2 [4], which is found abundantly in the nasal epithelium, on both ciliated and mucus-secreting goblet cells [37]. It is found that metformin increases the stability of ACE2 by its phosphorylation [4] in human endothelial cells and embryonic kidney cells, which leads to conformational and functional changes in the ACE2 receptor [4], thus leading to decreased binding between the ACE2 receptor and the binding domain of the SARS-CoV-2 receptor (RBD) [4]. Metformin contributes to reduced mortality rates due to optimized vascular health [31], better glycemic control [4,16,31,33], and decreased cytokine production [38] among diabetic patients infected with the COVID-19 virus [39]. In another retrospective study, the patient’s group treated with Metformin showed markedly decreased mortality rates [2.9% (3/104)] compared to the non-metformin treated group [12.3% (22/179)] (*p* = 0.01) [38]. In a retrospective cohort study, thirty-day mortality was lowest in SARS-CoV-2 residents taking metformin (12.6%, *n* = 12) compared with those on other diabetes medications (17.4%, *n* = 12), insulin (23.3%, *n* = 24), and no diabetes medications (22.7%, *n* = 108) [40]. Conversely, the relationship between the risk of COVID-19 and metformin therapy among patients with type 2 DM remains controversial, and future studies are needed [41]. A meta-analysis done by Nguyen et al. (2022) has found that Metformin administration is associated with decreased mortality rates among patients with diabetes T2 infected with COVID-19 [OR 0.54 95% CI 0.47–0.62] [12].

- Glucagon-like Peptide-1 Receptor Agonists (GLP-1RA)

**Figure 3:**
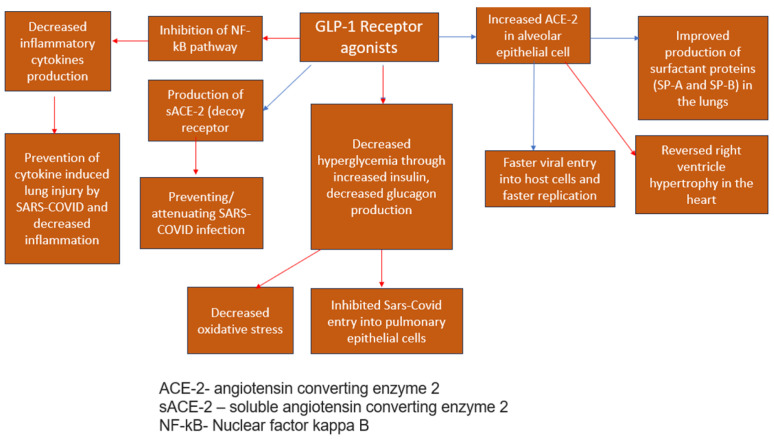
Antiviral effects of GLP1-RA agonsis [34,45]—blue arrows indicate stimulation (increase); red arrows indicate inhibition (decrease).

GLP-1RA agonists—Semaglutide, Exenatide, and Liraglutide—interfere with endogenous GLP-1 [42,43], an incretin hormone known for controlling glucose homeostasis [44]. Apart from their roles in glycemic modulation, these agents significantly reduce cardiovascular risks [42], induce weight loss [45], and prevent cytokine-induced lung injury by interfering with the NF-kB pathway [42,43,45] and exerting anti-inflammatory effects [44] (Figure 3), thus preventing the cytokine storm influenced by a hyperinflammatory response [46] and decreasing disease severity [42]. Haryanto et al. (2021) found that the use of GLP1-RA (liraglutide) was capable of stimulating the expression of pulmonary ACE2 [42,45], mainly expressed in alveolar epithelial cells, enterocytes, and blood vessels [42] upstream of the counter-regulatory RAS pathway [42], which exerts a negative effect on inflammatory and fibrotic processes [44] and can slow down the progression of acute respiratory distress syndrome (ARDS) [42,45], including the one caused by the SARS-CoV-2 infection [45]. Therefore, the immune-modulating impacts significantly control the increased immune responses among COVID-19 patients [42] and optimize patient outcomes [45]. The cardiovascular impacts linked to GLP-1 agonists improve the prognosis of COVID-19 diabetic patients [45,46] since they effectively reduce ventricular hypertrophy, optimizing cardiac functions and hypertension values [44]. In addition, GLP-1 agonists are found to increase surfactant protein production (SP-A, SP-B) [44,45] and thus, improve lung function [44]. Conversely, there is a minor concern regarding the ability of the drug to increase the expression of the Angiotensin-converting enzyme (ACE-2) which is used by SARS-CoV-2 to enter pulmonary epithelial cells [45]. Theoretically, it could help the viral spread and invade epithelial cells, although it is still unknown if this effect takes place in humans [45]. Rubino et al. (2020) explored the correlation between mortality rates and GLP-1 agonist administration among COVID-19 patients with diabetic complications. This study’s findings demonstrated the importance of GLP-1 agonists in managing diabetic patients infected with COVID-19. Conversely, due to limitations in observational designs, the study proposed further research to determine the causal link between GLP-1 and mortality rates among this patient population. Another meta-analysis by Nguyen et al. (2022) has found that the treatment with GLP-1 agonists is effective in decreasing mortality among diabetic T2 patients infected with COVID-19 [OR 0.51, 95% CI (0.37–0.69)] [12].

- Sulfonylureas (SU)

**Figure 4:**
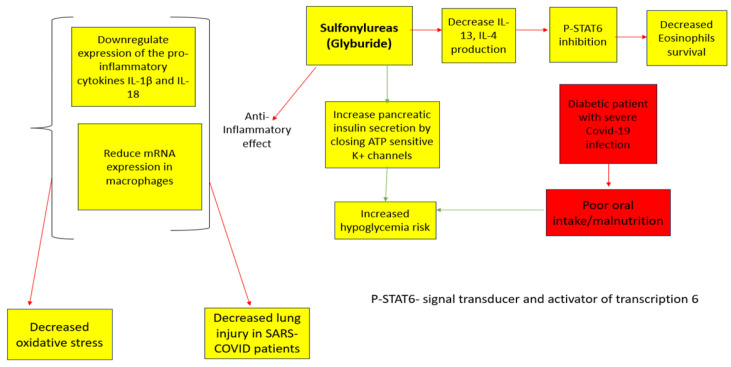
Sulfonylureas Anti-Viral Effects [12]. The red arrows indicate inhibition (decrease); green arrows indicate stimulation (increase).

This is a classification of antidiabetic drugs recognized for enhancing insulin secretion from the pancreatic beta cells [47] (Figure 4). Since the beginning of the COVID-19 pandemic, these drugs have been used due to their ability to improve glycemic control and decrease the expression of inflammatory cytokines, including the Tumor Necrosis Factor (TNF), Interleukin -4, interleukin 1B, and C-Reactive Protein (CRP) [48], thus reducing COVID-19-induced inflammation and lung damage [48]. Conversely, these regimens are related to severe hypoglycemia [1,48] which is commonly observed in COVID-19 patients due to poor oral intake [48]. Therefore, Sulfonylureas should be avoided in patients with a severe COVID-19 infection [1]. A meta-analysis done by Nguyen et al. (2022) has found the treatment with Sulfonylureas as mortality neutral [OR 0.92, 95% CI 0.83–1.01]; an important fact to consider regarding this result is the fact that Sulfonylureas are considered cardiovascular-neutral [12,48]. Thus, it is reasonable that they had no impact on mortality rates within COVID-19 patients, who have high rates of cardiovascular events resulting from increased inflammation and coagulation which were the primary drivers of intensive care unit admissions, mechanical ventilation requirements, and fatalities among COVID-19 patients [12].

- Dipeptidyl peptidase-4 inhibitors (DPP-4i)

**Figure 5:**
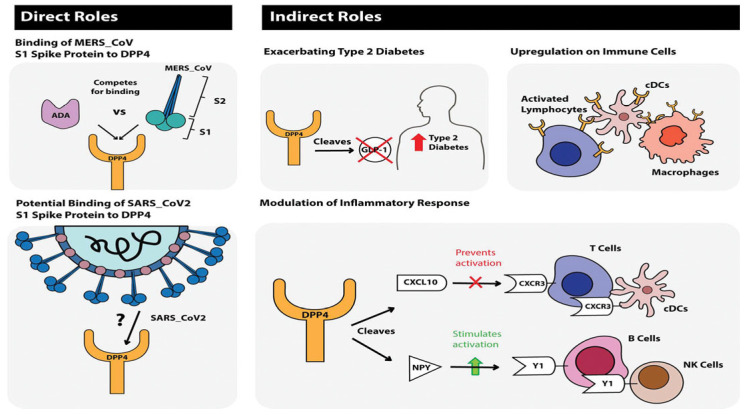
Journal of Diabetes, Volume: 12, Issue: 9, Pages: 649–658, First published: 11 May 2020, DOI: (10.1111/1753-0407.13052) [22].

Dipeptidyl peptidase-4 inhibitors are another group of antidiabetic drugs recognized for managing and controlling the effects of COVID-19 among diabetic patients [22]. This group of drugs is one of the most frequently prescribed anti-diabetic drugs, without serious adverse events [1]. Therapy with DPP-4i has proved neutral in terms of major adverse cardiac events in previous cardiovascular outcome trials [12]. The various effects of DPP-4i are achieved by inhibiting the DPP-4 enzyme, which normally degrades the incretin hormone (GLP-1) [1] (Figure 5) and is involved in the infection and spread of COVID-19 in the host [1], including the proliferation of T cells [22], NF-kB pathway enhancement [49], and thus, increased inflammatory cytokines production [50], B cell activation, and CD86 expression [12]. Therefore, it decreases the COVID-19-induced hyper-inflammatory state [12,22]. Gorrell et al. (2020) mention that DPP4 is also located on macrophages and classical dendritic cells (cDC) and is upregulated on activated lymphocytes [22]. Additionally, there is a predicted binding between the S1 Spike protein of SARS-CoV and DPP4 [22,51], potentially facilitating the infection of epithelial cells [22]. Conversely, a meta-analysis done by Nguyen et al. (2022) has found that treatment with DPP-4 inhibitors increases the mortality among diabetics infected with COVID-19 (OR 1.23, 95% CI 1.07–1.42) [12]. Another study found no difference in mortality rates (matched-analysis = odds-ratio: 0.94 [95% confidence interval: 0.69–1.28], *p*-value: 0.689) or any of the secondary outcomes (ICU admission, invasive ventilation, thrombotic events, or infectious complications) following treatment with DPP-4i [52]. Most observational studies have generated diverse outcomes [1,12]. These differences can be related to variances in patient populations, other underlying factors, and treatment procedures [14], hence the need for further research on this drug’s mechanism of protection against a severe COVID-19 infection course [50].

- Sodium-glucose cotransporter-2 Inhibitors (SGLT-2i)

**Figure 6:**
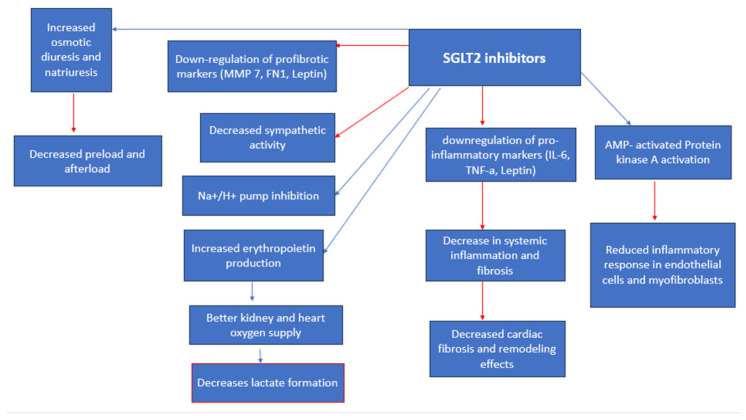
SGLT cardiorenal effects/anti-viral effects. Red arrows indicate inhibition (decrease); blue arrows indicate stimulation (increase).

The Sodium-glucose cotransporter-2 inhibitors (SGLT-2i) are classified as anti-diabetic drugs, which function by inhibiting the resorption of glucose and sodium in the proximal convoluted tubule of the kidney [53], thus improving glycemic control [54]. They are found to significantly improve the quality of life and reduce mortality rates among diabetes patients infected with COVID-19 [3], as well as have important renal protective [55] and cardiovascular benefits [5]. A meta-analysis by Nguyen et al. (2022) has found that treatment with SGLT-2i resulted in lower mortality rates (OR 0.60, 95% CI 0.40–0.88) [12]. SGLT-2i demonstrates the ability to decrease cardiovascular remodeling and stress by decreasing arterial stiffness by optimizing vasodilation and natriuresis [56]. Moreover, SGLT-2i have proven to decrease the expression of profibrotic and proinflammatory cytokines [53,56] (Figure 6) such as leptin, interleukin (IL)-6, and tumor necrosis factor (TNF) levels which are all contributors to cardiac inflammation [57]. In addition, SGLT-2i ameliorates oxidative stress [56,58,59] and reduces sympathetic activity [57], thus resulting in the downregulation of both systemic and adipose tissue inflammation [57]. Due to the emerging new COVID-19 strains [37] and increasing infection rates worldwide, there are economical clinical illustrations for the use of SGLT-2 inhibitors due to their importance in minimizing mortality rates [56,60]. It is important to note that the SGLT-2i’s ability to increase glucosuria [34], and thus, calorie loss, requires significant considerations since it is important to maintain an appropriate nutritional intake [1], especially in elderly and malnourished patients. SGLT2i treatment can result in diabetic ketoacidosis (DKA), especially in critically ill patients [53,61]. Moreover, SGLT-2i can result in a decreased glomerular filtration rate, which is required to be closely monitored during treatment [62]. Tuttle et al. (2022) assert that the slightly lower eGFR after the initiation of treatment with dapagliflozin (mean of approximately 3–5 mL/min per 1.73 m^2^) during hospitalization is expected, as the mechanism of action of the drug is to reduce glomerular hyperfiltration and is not associated with AKI or other adverse events [63]. Furthermore, osmotic diuresis can result in dehydration [1].

- Intestinal alpha-glucoside hydrolase inhibitor (AGI)—Acarbose

Acarbose is a complex oligosaccharide [64] that acts as a competitive, reversible inhibitor of pancreatic alpha-amylase and membrane-bound intestinal alpha-glucoside hydrolase [64], thus decelerating glucose absorption and diminishing concentrations of postprandial glucose in the bloodstream [65]. Furthermore, Acarbose could potentially induce weight loss through an increase in glucagon-like peptide-1 activity [64].

Li, Wei, et al. (2021) found that the utilization of acarbose, either as a single intervention or in conjunction with metformin, during the treatment of COVID-19 patients with type 2 diabetes mellitus (T2DM), is linked to a reduction in mortality rates [65]. Nguyen et al. (2022) found that AGI is mortality neutral (OR 0.61, 95% 0.26–1.45) when comparing between medication users and nonusers [12]. Acarbose stands as an economical therapeutic option that is widespread, particularly in China [65]. Given the individual benefits observed with metformin and acarbose, the potential synergistic or additive effects of using both substances concurrently are worth considering [65]. However, the efficacy of combining metformin and acarbose as a treatment strategy for diabetics infected with COVID-19 should be determined in future studies [65].

- Thiazolidinediones (TZD)-Pioglitazone

**Figure 7:**
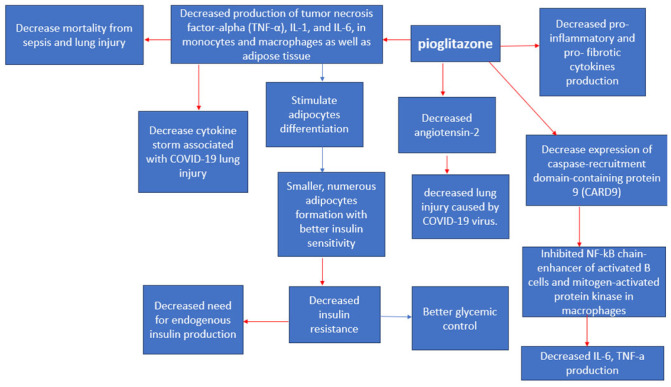
Pioglitazone anti-viral effects [66]. Red arrows indicate inhibition (decrease); blue arrows indicate stimulation (increase).

This class of drugs might have a significant role in individuals with diabetes who are affected by COVID-19 [21] as they experience an excessive inflammatory reaction driven by the SARS-CoV-2 virus [53], which leads to the cytokine storm syndrome [46]. To counteract this heightened inflammation, peroxisome proliferator-activated receptor-γ (PPARγ) agonists have been shown to reduce the production of different pro-inflammatory cytokines such as the tumor necrosis factor-alpha (TNF-a), IL-1, and IL-6 in monocytes and macrophages [21] (Figure 7). Particularly, PPAR-y agonists have demonstrated the ability to lower the expression of the caspase-recruitment domain-containing protein 9 (CARD9) [66], subsequently inhibiting the activation of pathways like the nuclear factor k-B (NF-kB) in B cells and mitogen-activated protein kinase in macrophages [66]. Notably, adipose tissue actively contributes to inflammation by releasing various proinflammatory proteins [21], including TNF-a, IL-6, and monocyte chemoattractant protein-1 (MCP-1) [67]. Animal studies have shown that pioglitazone can suppress the generation of these inflammatory cytokines in adipose tissue [67]. Additionally, in animal models, pioglitazone has been observed to reduce mortality associated with sepsis and lung injury by decreasing the production of inflammatory cytokines in omental tissue [67]. This significant reduction in cytokine production has prompted researchers to consider its potential role in mitigating the cytokine storm associated with COVID-19 [66,67]. In addition, TZDs stimulate adipocyte differentiation [21,68], preferentially generating more numerous, smaller adipocytes that are more insulin-sensitive [68]. Nevertheless, it is important to note that the lack of direct comparability between human and mouse responses could limit the applicability of these findings in a clinical context [67]. Pioglitazone is known to enhance the expression of angiotensin converting enzyme 2 (ACE2) [67,69] which could play a dual role in the context of COVID-19 [67]. On one hand, it might increase the vulnerability to infection, given that SARS-CoV-2 uses ACE2 as a co-receptor to infiltrate alveolar cells [67]. Conversely, these drugs could have a protective aspect by lowering angiotensin II levels [67,69], which guards against acute lung injury [67]. Currently, there is no substantiated proof indicating that pioglitazone stimulates ACE2 upregulation in alveolar cells [67]. Instead, evidence from animal research suggests an elevation of ACE2 in insulin-sensitive tissues [69], potentially serving as a safeguard against lung injury [67]. Nyland et al. (2021) found that patients treated with pioglitazone showed a relative reduction of 29.2% in hospital admissions but did not have significant reductions in respiratory complications or mortality rates [46]. Another study by Nguyen et al. (2022) concluded that Pioglitazone is mortality neutral [OR 0.90 (95% CI 0.71–1.14)] when comparing medication users and nonusers [12].

## 4. Discussion

Since 2019, when COVID-19 emerged [45], diabetes has become a major risk factor for a severe infection course [10] because it impairs the immune system’s function [10] and induces hypercoagulability [1]. Additionally, chronic hyperglycemia induces alveolar hyperpermeability [12] and causes vascular endothelial cell damage by diminishing nitric oxide, thus inducing vasoconstriction [12] and increasing the risk of thromboembolism and cardiorespiratory failure [1]. Furthermore, the COVID-19 infection enhances insulin resistance [70], therefore causing hyperglycemia deterioration [1]. Altogether, this leads to challenging infection control in diabetics with COVID-19 [71]. The high risk of diabetic patients developing complications following infection with COVID-19 [6] resulted in significant research [12] on the anti-diabetic drugs’ effect on mortality rates among diabetics with COVID-19 infection [71]. Researching this topic is complicated due to the increasing prevalence of obesity [72], hypertension, cardiovascular disease [73], and the aging population worldwide [1,10] as these factors affect the disease’s progression [71], and therefore, the mortality rates. The drugs that were associated with increased mortality rates among diabetics with COVID-19 infection are insulin (OR 1.70, 95% CI 1.33–2.19) and DPP-4i (OR 1.23, 95% CI 1.07–1.42), while Metformin (OR 0.54, 95% CI 0.47–0.62), GLP-1RA (OR 0.51, 95% CI 0.37–0.69), and SGLT-2i (OR 0.60, 95% CI 0.40–0.88) resulted in decreased mortality rates, mainly through the reduction of inflammatory cytokine production and cardioprotective and hypoglycemic effects [12]. Sulfonylureas (OR 0.92, 95% 0.83–1.01), Thiazolidinediones (OR 0.90, 95% CI 0.71–1.14), and AGI (OR 0.61, 95% 0.26–1.45) are found to be mortality neutral [12]. These findings could significantly influence the approach to managing diabetes patients in outpatient settings during the COVID-19 pandemic [1,12], as they offer valuable insights to healthcare professionals when selecting suitable glucose-lowering treatments for these patients to mitigate the risk of in-hospital mortality [12], notably by advocating for the prescription of metformin, GLP-1RA, and SGLT-2i unless contraindicated [1,12]. Conversely, caution is advised when considering long-term insulin therapy or DPP-4i [12,26].

## 5. Limitations

Several inherent limitations should be acknowledged, including disparities in vaccination status [74], variations in COVID-19 strains in different countries [10], different hospitalization protocols [12], and variable healthcare infrastructure [19], which were not examined [60]. Although this review aims to provide insights into the potential efficacy of various antidiabetic drugs in reducing mortality during the COVID-19 pandemic, it is important to exercise caution as some findings require further research and clarification [12]. Moreover, many of the studies reviewed herein are observational; thus, the potential for confounding factors and biases is inherently introduced [3]. To counteract this risk, we chose studies that reported a low risk of bias [odds ratio (95% CI)] and studies that included large population samples [12]. These studies used a meta-regression, subgroup analysis, and sensitivity analysis to confirm the robustness of their findings. Outliers were identified and removed; the heterogeneity of all remaining studies drastically decreased without a significant change in the OR (all *p* > 0.05), indicating that the pooled odds ratio still reflected the actual effect size [12]. In addition, the publication bias can skew the overall assessment of the literature, wherein studies with statistically significant findings are more likely to be published [12].

## 6. Conclusions

The potential of antidiabetic drugs to influence mortality outcomes among diabetic patients infected with COVID-19 is largely variable. There is a need for substantial research on the exact antiviral mechanisms of antidiabetic medications, their adverse effects, their possible drug–drug interactions, and the contraindications to use in different patient populations. Conversely, the dynamic landscape of the pandemic, the variability in study methodologies, and the potential for biases in observational studies demonstrate the challenges of developing definitive conclusions [12].

## Data Availability

Not applicable.
